# Sodium glucose co-transport 2 inhibitors in the treatment of type 2 diabetes mellitus: a meta-analysis of randomized double-blind controlled trials

**DOI:** 10.1186/1472-6823-13-58

**Published:** 2013-12-17

**Authors:** Asres Berhan, Alex Barker

**Affiliations:** 1 Hawassa University College of Medicine and Health Sciences, P. O. Box: 1560, Hawassa, Ethiopia; 2 Veteran Affairs Medical Center, Iron Mountain, Michigan, USA

**Keywords:** Canagliflozin, Dapagliflozin, Empagliflozin, Ipragliflozin, Meta-analysis, SGLT2 inhibitors, Type 2 diabetes

## Abstract

**Background:**

The discovery of sodium-glucose co-transporter 2 (SGLT2) inhibitors, with a novel mechanism independent of insulin secretion or sensitization, bring about a new therapeutic approach to the management of type 2 diabetes mellitus. The aim of this meta-analysis was to evaluate the safety and efficacy of SGLT2 inhibitors at different doses in randomized double blind clinical trials.

**Methods:**

This meta-analysis was conducted by including randomized double-blind controlled trials of SGLT2 inhibitors in patients with type 2 diabetes irrespective of their antidiabetic drug exposure history but with an inadequate glycemic control. All the effect sizes were computed using the random effects model. Standardized mean differences (SMDs) and odds ratios (OR) were computed for continuous and dichotomous variables, respectively. Additional analyses like sensitivity analysis, subgroup analysis and meta-regression were also performed.

**Results:**

The pooled analyses demonstrated a significant reduction in mean changes in Hemoglobin A1c (HbA1c) (SMD = −0.78%, 95% CI, -0.87 to −0.69), fasting plasma glucose (FPG) (SMD = −0.70 mg/dl, 95% CI, -0.79 to −0.61), body weight (overall SMD = −0.59 kg, 95% CI, -0.65 to −0.52) and blood pressure from baseline with SGLT2 inhibitors based therapy. Consistently a significant number of patients treated with SGLT2 inhibitors achieved HbA1c < 7% (OR = 2.09, 95% CI, 1.77 to 2.46). SGLT2 inhibitors based therapy was associated with adverse events like genital and urinary tract infections.

**Conclusion:**

All studied doses of SGLT2 inhibitors, either as monotherapy or in combination with other antidiabetic agents, consistently improved glycemic control in patients with type 2 diabetes. However, a small percentage of patients suffer from genital and urinary tract infections.

## Background

The persistent hyperglycemia in patients with type 2 diabetes mellitus is strongly associated with microvascular and macrovascular complications [1]. Chronic hyperglycemia is postulated to contribute to the continuous loss of pancreatic β-cells and the impairment of insulin secretion [2]. Microvascular complications like diabetic retinopathy, nephropathy, and neuropathy are major causes of new cases of blindness and renal insufficiency [3]. Moreover, in type 2 diabetes macrovascular complications, including coronary heart disease and stroke, are major causes of morbidity and mortality [4]. According to a prospective study, in patients with type 2 diabetes, a 1% increase in HbA1c was associated with 20% to 30% increase in mortality or cardiovascular events [5].

Intensive glycemic control in patients with type 2 can delay the onset and progression of the early stages of diabetic microvascular complications [6]. In the UKPDS, a reduction in mean HbA1c was associated with reductions in both microvascular and macrovascular complications [1]. But the risk reduction of myocardial infarction, stroke, and heart failure was relatively low [1]. Furthermore a meta-analysis of randomized controlled trials reported that an intensive glycemic control resulted in no significant effect on events of stroke or all-cause mortality [7]. As a result, researchers recommend a multidisciplinary approach to the management of the cardiovascular risk factors in patients with type 2 diabetes [8].

Currently many antidiabetic agents are available with a variety of chemical groups and site of actions. Most of these agents act by either improving the insulin sensitivity or by enhancing insulin secretion. Nevertheless, as the pancreatic β-cells function continues to decline, failure of therapy to achieve adequate glycemic control is inevitable. The cumulative incidence of monotherapy failure was 15% with rosiglitazone, 21% with metformin, and 34% with glyburide after 5 years of therapy [9]. On the other hand, agents like insulin, sulphonylureas, and thiazolidinediones are associated with significant safety concerns such as weight gain and hypoglycemic events [9,10].

The discovery of SGLT2 inhibitors, with novel mechanism independent of insulin secretion or sensitization, may possibly expand the armamentarium in the battle against type 2 diabetes mellitus. SGLT2 plays an important role in the kidneys and is responsible for most renal glucose re-absorption in the proximal convoluted tubule [11]. Currently there are a number of SGLT2 inhibitors that are under development or in clinical trials [12]. Prior meta-analyses had established the safety and efficacy of SGLT2 inhibitors as a group [13,14]. However, recently published randomized clinical trials reported more frequent adverse events with SGLT2 inhibitors and no significant difference in the proportion of patients achieving HbA1c levels <7.0% as compared to placebo treated [15,16]. Thus, the primary aim of this meta-analysis is to determine the safety and efficacy of SGLT2 inhibitors alone or in combination with other anti-diabetic drugs relative to placebo or placebo with other anti-diabetic drugs by including both previously and recently published randomized double blind clinical trials.

## Methods

### Search strategy

Electronic based literature search was conducted in the databases of MEDLINE, HINARI, EBASE and The Cochrane Library by both authors (AB and AB). The literature search was further strengthened by searching relevant articles from the reference list of retrieved articles. During searching the following search terms were alternatively combined using the Boolean logic (AND, OR and NOT): sodium glucose co-transport (SGLT) inhibitors, dapagliflozin, canagliflozin, ipragliflozin, empagliflozin, sergliflozin etabonate, remogliflozin etabonate, tofogliflozin and type 2 diabetes.

### Inclusion criteria and study selection

The predetermined study inclusion criteria were: (1) randomized double-blind controlled trials of SGLT2 inhibitors in patients with type 2 diabetes mellitus; (2) studies which recruited patients with type 2 diabetes irrespective of their antidiabetic drug exposure history (naïve or drug experienced) but with an inadequate glycemic control (HbA1c ≥7.0); (3) studies written in English and (4) studies with a minimal duration of therapy for 12 weeks. The study selection of the retrieved literature was conducted in two steps: First, all the retrieved literature titles and abstracts were reviewed and then grouped either under “eligible for full document review” or “ineligible for full document review”. Second, all literatures that were grouped under “eligible for full document review” were reviewed in detail and then grouped as “eligible for the meta-analysis” or “ineligible for the meta-analysis”.

### Data extraction and quality assessment

Data extraction from the selected studies was conducted by both authors independently with the same data extraction template. Standard Excel spreadsheets were used for the data extraction. The following information was abstracted from the included studies: name of the first author, year of publication, sites of the study, the study design, duration of therapy, antidiabetic drugs used by the patients before they were recruited in the studies, antidiabetic drugs used in combination with SGLT2 inhibitors, dose, change in HbA1C(%) from baseline, number of patients with HbA1c < 7.0%, change in FPG, change in body weight, change in blood pressure, number of patients with adverse events, And number of patients who discontinued medication due to adverse events, experienced serious adverse events, experienced hypoglycemia, experienced urinary tract infection, and experienced genital tract infection.

Risk of bias in every of the included studies was assessed by the Cochrane risk of bias assessment tool. The predefined key domains were: random sequence generation, allocation concealment, blinding of participants and personnel, blinding of outcome assessment, incomplete outcome data, selective reporting and other bias. Based on the included articles, each domain was judged as “low risk of bias” or “unclear risk of bias” or “high risk of bias”.

### Data synthesis and statistical analysis

Before the pooled analyses were conducted, some statistical transformations and unit conversions were performed. In case of continuous variables, where the standard deviation (SD) was not reported in the included studies, we computed the SD from standard errors (SE), 95% confidence intervals (CI) or P-values. When the value of FPG was reported in mmol/L, it was converted to mg/dl using an online converter [17].

All effect sizes in this meta-analysis were computed using the random effects model. SMDs and the 95% CIs were computed for the changes in HbA1C (%), FPG, body weight, and blood pressure from baseline using the inverse variance method (IV). For dichotomous variables (adverse events, discontinuation of medication due to adverse events, serious adverse events, hypoglycemic events, urinary tract infection, and genital tract infection) ORs and 95% CIs were computed with Mantel-Haenszel method (M-H). When the 95% CI does not include zero for the SMDs and one for ORs, it was considered as statistically significant. Sensitivity analysis was performed by removing a study with a specific dose from the analysis at a time to evaluate the stability of the pooled values.

The consistency of the included studies was evaluated by the heterogeneity test (I2 statistics); when the value of I2 is greater than or equal to 50%, the included studies were considered as statistically inconsistent. To identify the possible sources of heterogeneity subgroup analysis and meta-regression were conducted. Publication bias was assessed by funnel plots and by funnel plot asymmetry test (Egger’s test). All the statistical analyses were performed with OpenMetaAnalyst software and Review Manager (RevMan) Version 5.1 software. We reported the meta-analysis by following the PRISMA checklist.

## Results

**Figure 1:**
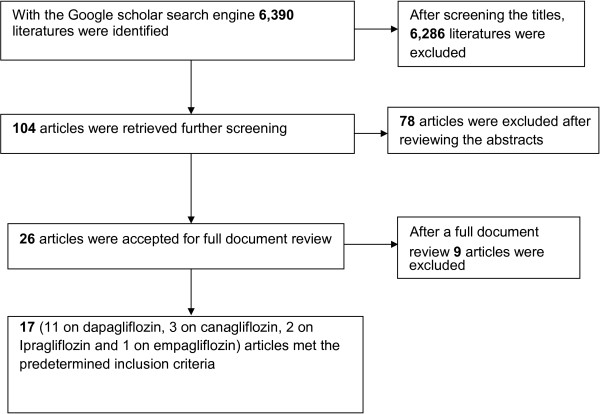
Study selection flow diagram.

Using the Google scholar search engine about 6,390 literatures on SGLT2 inhibitors were identified. One hundred and four articles were retrieved. After reviewing the abstracts of all the retrieved articles 26 articles were selected for full document review. Then, following the full document review of the 26 articles, 17 fulfilled the predetermined inclusion criteria. Eleven of studies were on dapagliflozin [15,18-27], 3 on canagliflozin [16,28,29], 2 on ipragliflozin [30,31], and the remaining one was on empagliflozin [32] (Figure 1). A total of 4,811 patients with type 2 diabetes received one of the SGLT2 inhibitors alone (2,686) or in combination with other antidiabetic drugs (2,125) and 1,921 received placebo (887) or placebo with other antidiabetic drugs (1,034) (Table 1). The risk of bias assessment in the included individual studies did not demonstrate the presence of bias in randomization, blinding or reporting. Similarly, funnel plot did not also show the existence of publication bias.

**Table 1:** Summary of the included studies in the meta-analysis

**Author**	**Year**	**Duration (wk)**	**Drug before**	**Background regimen**	**Group 1 (n)**	**Group 2 (n)**	**Group 3 (n)**	**Group 4 (n)**	**Group 5 (n)**	**Group 6 (n)**
Rosenstock J et al S1 [28]	2012	12	Metformin	Metformin	PBO (65)	CANA 50 mg QD (64)	CANA 100 mg QD (64)	CANA 200 mg QD (65)	CANA 300 mg QD (64)	CANA 300 mg BID (64)
Yale JF et al [29]	2013	26	N/R	N/R	PBO (90)	CANA 100 mg QD (90)	CANA 300 mg QD (89)			
Stenlof K et al [16]	2013	26	Diet and exercise	-	PBO (192)	CANA 100 mg QD (195)	CANA 300 mg QD (197)			
Ferrannini E et al S1 [32]	2013	12			PBO (82)	EMPA 5 mg QD (81)	EMPA 10 mg QD (81)	EMPA 25 mg QD (82)		
Fonseca VA et al [31]	2012	12	N/R	-	PBO (69)	IPRA 12.5 mg QD (70)	IPRA 50 mg QD (67)	IPRA150 mg QD (68)	IPRA 300 mg QD (68)	
Wilding JPH et al S1 [30]	2013	12	Metformin	-	PBO (66)	IPRA 12.5 mg QD (69)	IPRA 50 mg QD (68)	IPRA 150 mg QD (67)	IPRA 300 mg QD (72)	
Wilding JPH et al S2 [18]	2009	12		Insulin	PBO (19)	DAPA 10 mg QD (23)	DAPA 20 mg QD (23)			
Bailey CJ et al S1 [19]	2012	24	Naïve	-	PBO (68)	DAPA 1 mg QD (72)	DAPA 2.5 mg QD (72)	DAPA 5.0 mg QD (66)		
Ferrannini E et al S2 [20]	2010	24	Naïve	-	PBO (75)	DAPA 2.5 mg QD (65)	DAPA 5.0 mg QD (64)	DAPA 10 mg QD (70)		
Henry RR et al [21]	2012	24	Naïve	Metformin	PBO 1 (201)	DAPA 5 mg QD (194)	DAPA 10 mg QD (211)	PBO 2 (208)		
Strojek K et al [22]	2011	24	Glimepiride	Glimepiride	PBO (145)	DAPA 2.5 mg QD (154)	DAPA 5 mg QD (142)	DAPA 10 mg QD (151)		
Bailey CJ et al S2 [23]	2010	24	Metformin	-	PBO (137)	DAPA 2·5 mg QD (137)	DAPA 5 mg QD (137)	DAPA 10 mg QD (135)		
Bolinder J et al [24]	2012	24	N/R	Metformin	PBO (91)	DAPA 10 mg QD (88)				
Rosenstock J et al S2 [25]	2012	24	Pioglitazone	Pioglitazone	PBO (139)	DAPA 5 mg QD (141)	DAPA 10 mg QD (140)			
Kaku K et al [5]	2013	12	Naïve	-	PBO (54)	DAPA 1 mg QD (59)	DAPA 2.5 mg QD (56)	DAPA 5 mg QD (58)	DAPA 10 mg QD (52)	
Wilding JPH et al S3 [26]	2012	24	Insulin	Insulin	PBO (166)	DAPA 2.5 mg QD (179)	DAPA 5 mg QD (185)	DAPA 10 mg QD (173)		
List JF et al [27]	2009	12	Naïve	-	PBO (54)	DAPA 2.5 mg QD (59)	DAPA 5 mg QD (58)	DAPA 10 mg QD (47)	DAPA 20 mg QD (59)	DAPA 50 mg QD (56)

*n* number of patients, *N/R* not reported, *QD* once daily, *BID* twice daily, *PBO* placebo, *CANA* canagliflozin, *EMPA* empagliflozin, *IPRA* ipragliflozin, *DAPA* dapagliflozin.

**Figure 2:**
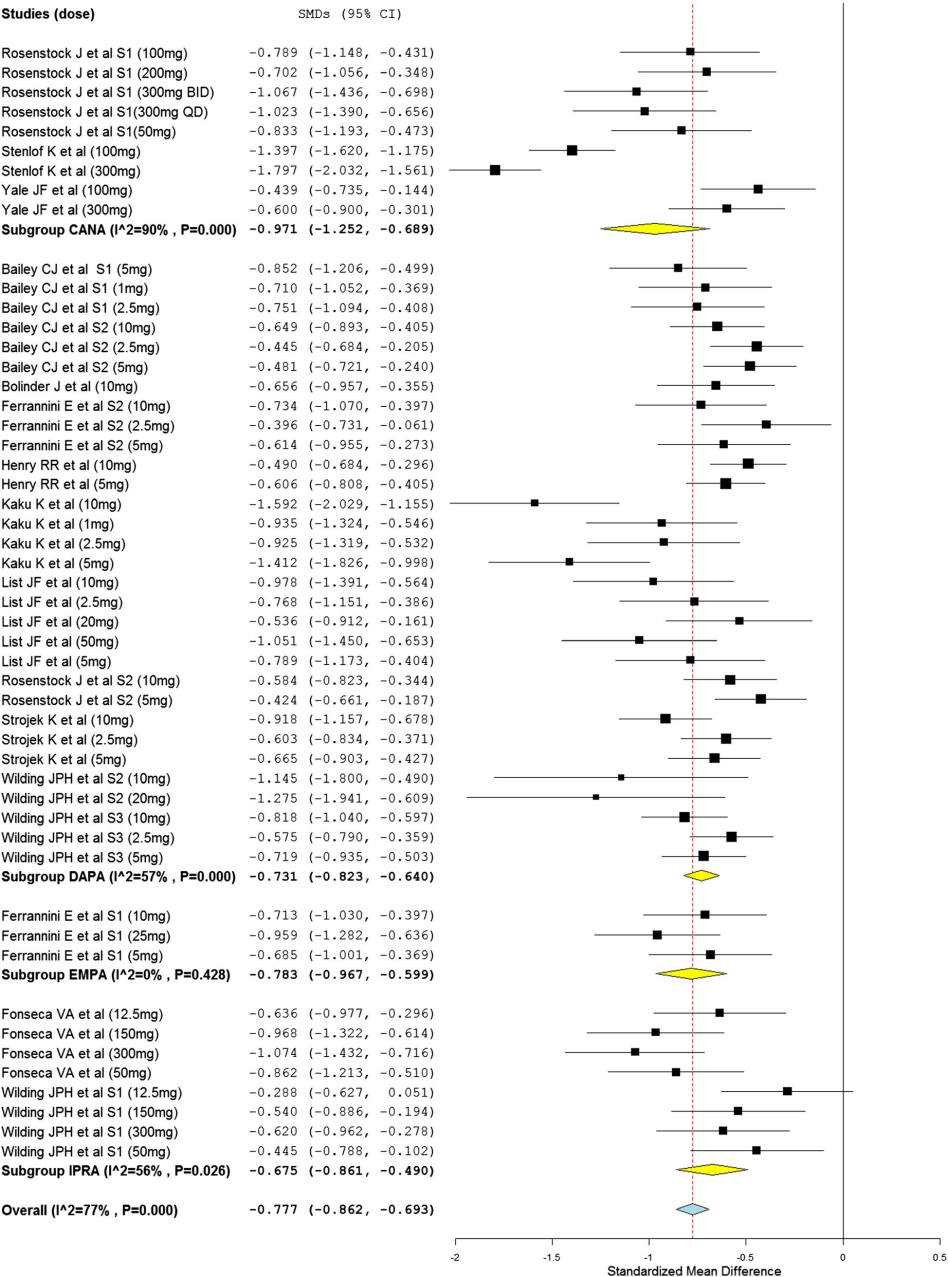
Standardize mean difference of the change in HbA1c from baseline.

As presented in Figure 2, the pooled analysis of the mean change in HbA1c from baseline established a significant reduction in patients who were treated with SGLT2 inhibitors than placebo treated patients (overall SMD = −0.78; 95%CI, -0.86 to −0.69). All the SGLT2 inhibitors included in the meta-analysis, canagliflozin (subtotal SMD = −0.97; 95%CI, -1.25 to −0.69) dapagliflozin (subtotal SMD = −0.73; 95%CI, -0.82 to −0.64), ipragliflozin subtotal SMD = −0.68; 95%CI, -0.861 to −0.490) and empagliflozin subtotal SMD = −0.78; 95%CI, -0.967 to −0.599), demonstrated the significant reduction in HbA1c. The reduction in HbA1c appears more prominent in canagliflozin treated patients. However, heterogeneity testing revealed the presence of a considerable heterogeneity among the studies on canagliflozin (I2 = 90%) and a moderate heterogeneity among studies on dapagliflozin (I2 = 57%) and ipragliflozin (I2 = 56%).

Subgroup analysis based on the doses of SGLT2 inhibitors and the type of regimen (SGLT2 inhibitors monotherapy vs SGLT2 inhibitors in combination with other antidiabetic drugs) and meta-regression using duration of therapy and the doses of SGLT2 inhibitors as a covariates did not show a significant difference in HbA1c change from baseline. On the other hand sensitivity analysis confirmed the stability of the overall SMD when any of the studies with a specific dose removed from the analysis. The overall SMD ranged within −0.75 to −0.79%.

**Figure 3:**
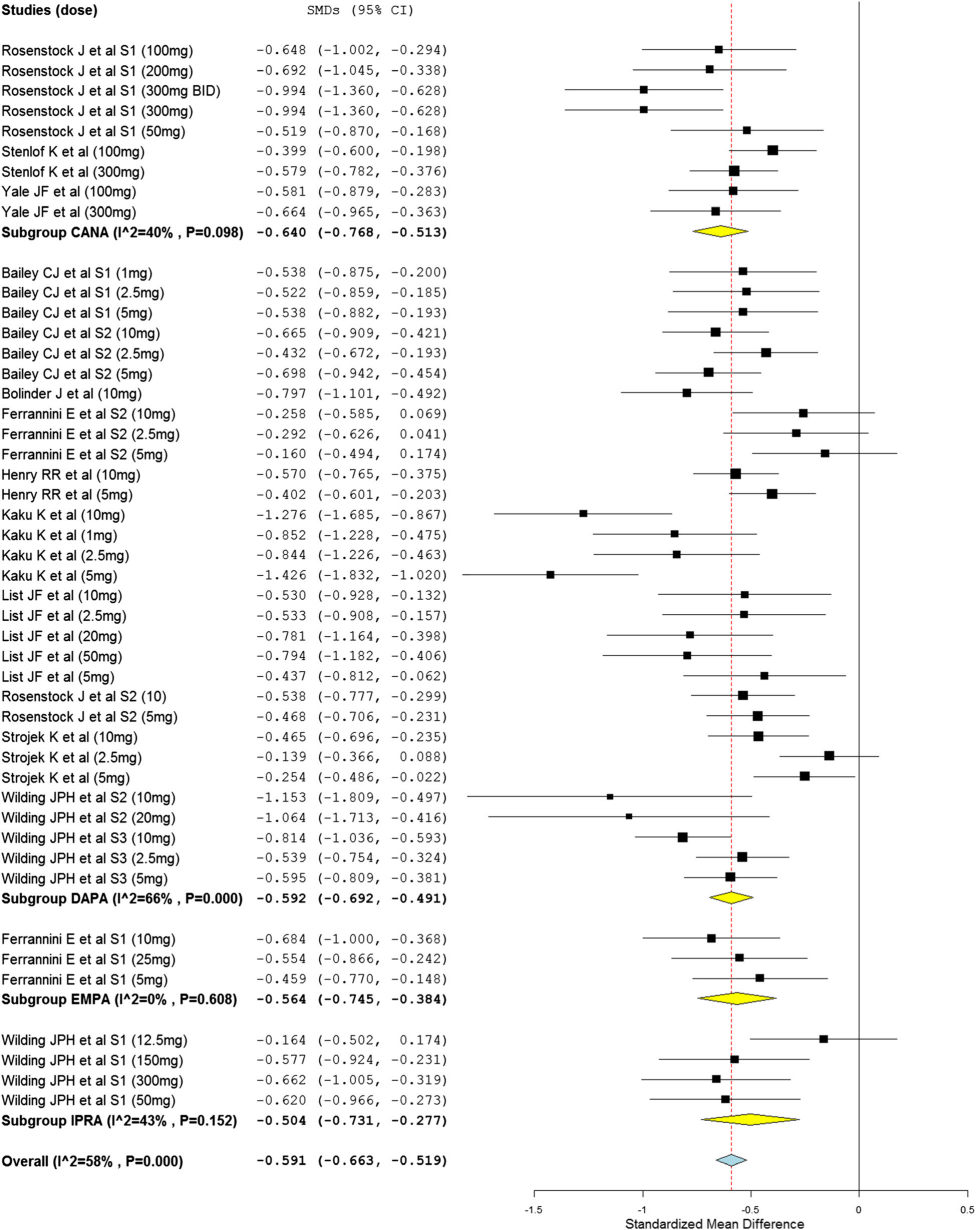
Standardize mean difference of the change in body weight from baseline.

In support of the above analysis, the odds of SGLT2 inhibitors treated patients who achieved HbA1c < 7.0% were more than two folds of placebo treated groups (overall OR = 2.09; 95% CI, 1.77 to 2.46). Similarly, the mean FPG levels (overall SMD = −0.70 mg/mL, 95% CI, -0.79 to −0.61) and mean body weight (overall SMD = −0.59 kg; 95% CI, −0.66 to −0.52) of patients who were treated with SGLT2 inhibitors were significantly decreased from baseline compared to placebo treated patients (Figure 3). Furthermore, treatment with SGLT2 inhibitors was significantly associated with a reduction in both systolic (overall SMD = −0.27 (mmHg; 95% CI, -0.34 to −0.20) and diastolic (overall SMD = −0.24, 95% CI, -0.30 to −0.17) blood pressure from baseline. Most of the individual studies did not show the significant association of SGLT2 inhibitors with an increase in HDL cholesterol level from baseline. However, the overall SMD demonstrated a significant increase in HDL cholesterol level in patients who were treated with SGLT2 inhibitors (overall SMD = 0.21 mg/dl; 95% CI, 0.09 to 0.33). The change in the level of LDL cholesterol from baseline in SGLT2 inhibitors treated groups was not different from placebo treated groups (overall SMD = 0.07 mg/l; 95% CI, -0.01 to 0.14).

**Figure 4:**
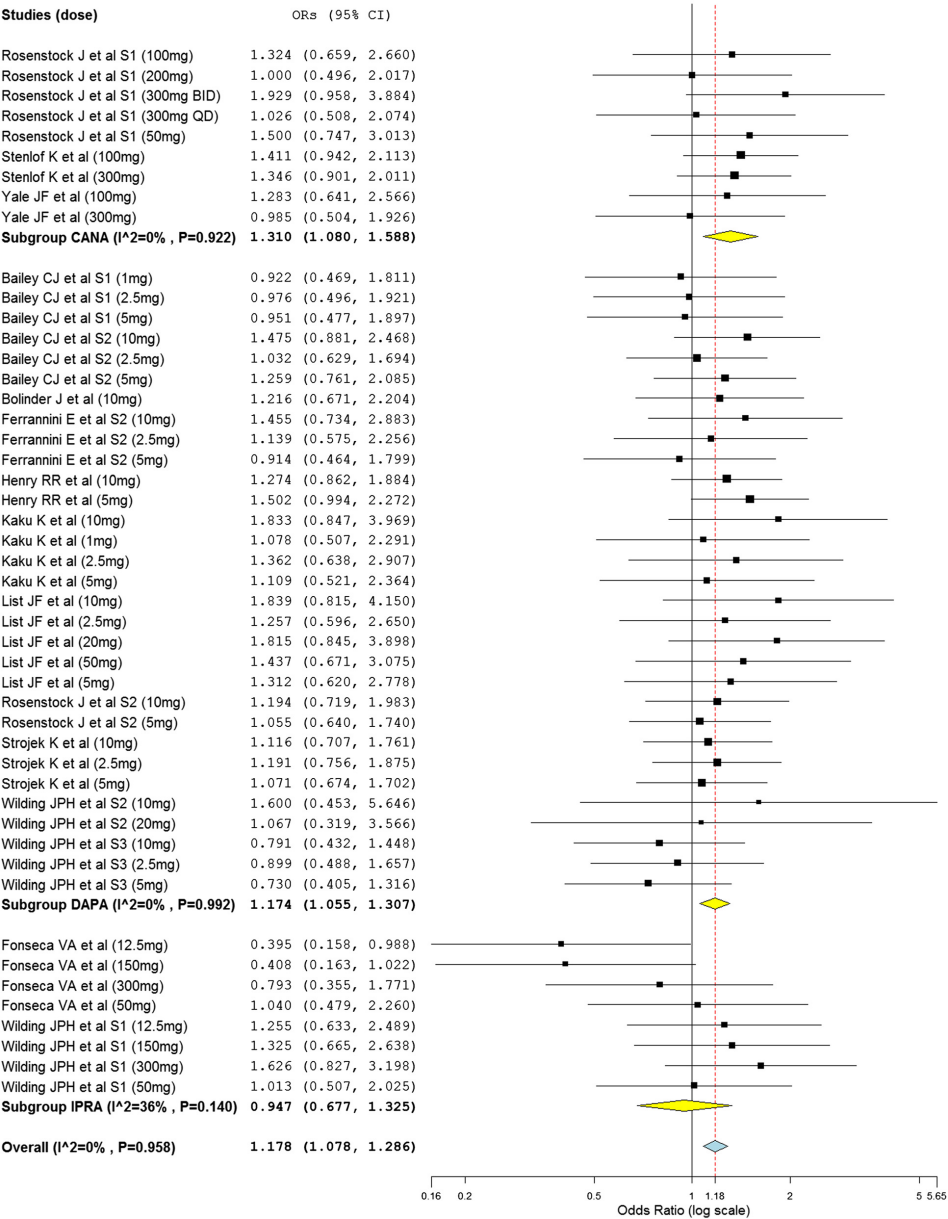
Mantel-Haenszel odds ratio of patients who experienced any adverse events.

**Figure 5:**
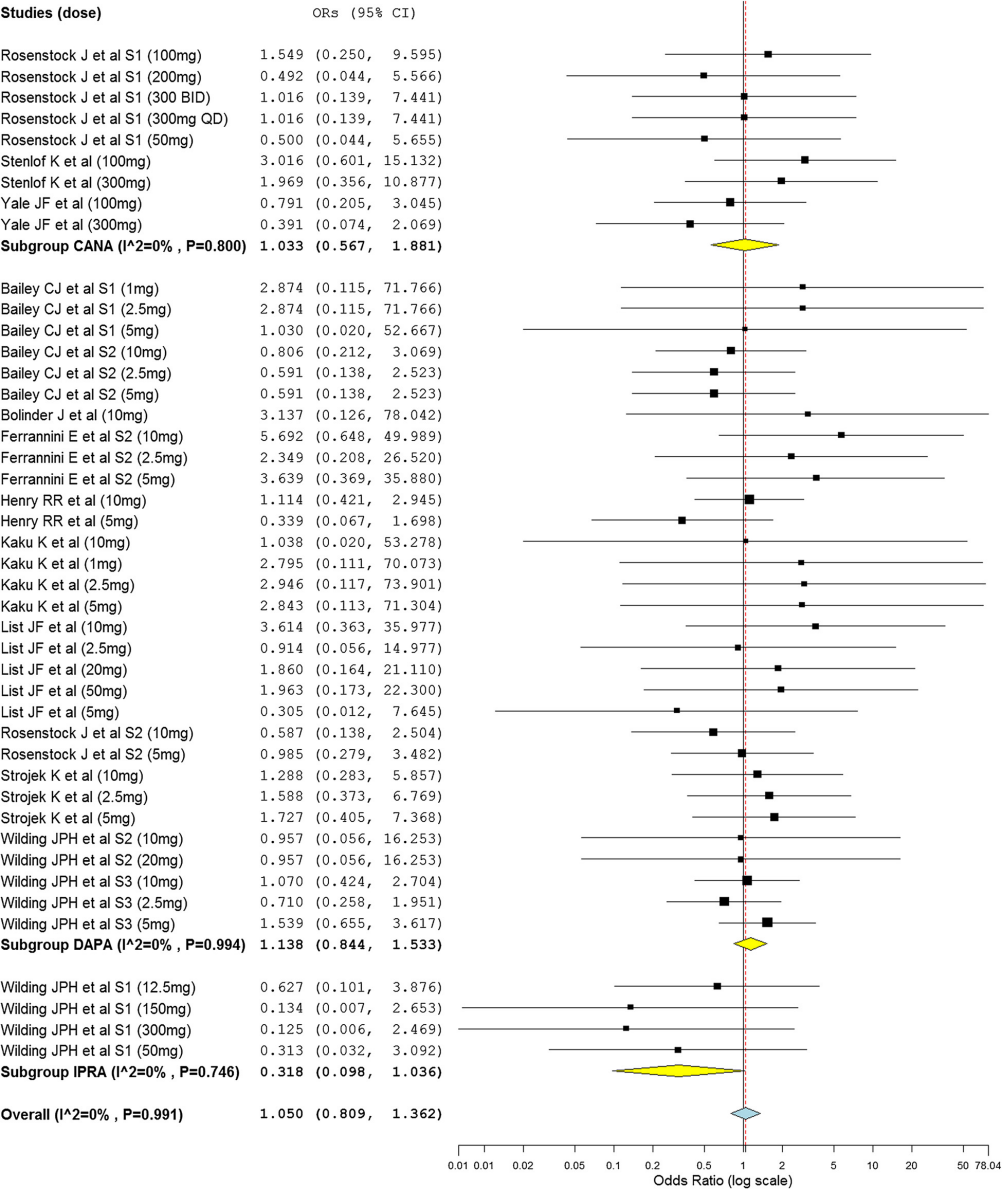
Mantel-Haenszel odds ratio of patients who discontinued the treatment due to adverse events.

Even though the SGLT2 inhibitors with all doses did not show association with adverse events, the overall OR revealed the significant association of SGLT2 inhibitors with adverse events (overall OR = 1.18; 95% CI, 1.08 to 1.29) (Figure 4). The subtotal ORs in the subgroups of canagliflozin (subtotal OR = 1.31; 95% CI, 1.08 to 1.59) and dapagliflozin (subtotal OR = 1.17; 95% CI, 1.05 to 1.31) showed significant association with adverse events. Whereas the subtotal ORs in the subgroups of ipragliflozin was not statistically significant (OR = 0.95; 95% CI, 0.677 to 1.325). Dapagliflozin (subtotal OR = 3.07; 95% CI, 2.32 to 4.05) and canagliflozin (subtotal OR = 3.42; 95% CI, 1.86 to 6.28) were associated with genital tract infections. Dapagliflozin was also associated with urinary tract infection (subtotal OR = 1.32; 95% CI, 1.06 to 1.63). Nevertheless the number of patients who were treated with SGLT2 inhibitors and experienced serious adverse events was not different from placebo treated groups (overall OR = 0.83; 95% CI, 0.65 to 1.05). Similarly the number of patients who experienced hypoglycemic events during the study periods was not different from placebo treated groups (overall OR = 1.16; 95% CI, 0.94 to 1.43). As presented in Figure 5, The odds of discontinuation of medication due to adverse events in the SGLT2 inhibitors treated patients was not significantly different from placebo treated patients (overall OR = 1.05; 95% CI, 0.81 to 1.36).

## Discussion

In agreement with prior meta-analyses [13,14] this meta-analysis demonstrated the significant improvement of glycemic control in patients with type 2 diabetes treated with SGLT2 inhibitors. Patients treated with SGLT2 inhibitors either as monotherapy or in combination with other antidiabetic agents significantly decreased HbA1c and FPG from baseline at all doses studied as compared to patients treated with placebo or placebo with other antidiabetic agents. A significantly large number of patients treated with SGLT2 inhibitors achieved HbA1c < 7% at the end of the study period of the included studies. The reduction of HbA1c from baseline with SGLT2 inhibitors may reflect the long term mean glycemic control for the previous 2 to 3 months; while, FPG indicates the glycemic control on the day of the visit [33,34].

On top of this, the meta-regression and subgroup analysis did not demonstrate a significant change in the efficacy of SGLT2 inhibitors depending on the duration of therapy and the doses SGLT2 inhibitors studied. Accordingly, the glycemic control with SGLT2 inhibitors does not seem to decline when the duration of therapy gets longer. On the other hand, the increase in doses of SGLT2 inhibitors was not accompanied by an increase in the efficacy of SGLT2 inhibitors. A phase 3 long-term extension dapagliflozin study (102 weeks), reported the sustained reductions in HbA1c and FPG level [35]. However, the finding of the meta-regression should not be taken as a confirmation of the long term efficacy and safety of SGLT2 inhibitors at all doses. The included studies in the meta-regression reported the change in HbA1c and FPG level after a duration of therapy not more than 26-weeks (Table 1).

Treatment with SGLT2 inhibitors was associated with a significant reduction in body weight, systolic and diastolic blood pressure. Therapy with SGLT2 inhibitors was associated with a rise in HDL cholesterol level without a significant change in LDL cholesterol level. Provided that most of the antidiabetic agents are associated with weight gain [9,10] and the vast majority of patients with type 2 diabetes are overweight or obese [36], the introduction of SGLT2 inhibitors with an insulin independent mechanism could have a pivotal role in the management of type 2 diabetes. The drop in blood pressure and the rise in HDL cholesterol level with SGLT2 inhibitor therapy could even make SGLT2 inhibitors more promising. This is because; in patients with type 2 diabetes the major cause of morbidity and mortality is attributed to cardiovascular diseases [4,37]. But SGLT2 inhibitors long term effects on cardiovascular outcomes is uncertain. Moreover, in this study, the meta-analyses of change in blood pressure and cholesterol levels were not controlled to change in body weight. Since body weight reduction was strongly associated with a drop in blood pressure and a change in cholesterol levels [38,39], the changes in blood pressure and cholesterol levels in SGLT2 inhibitors treated groups could be mediated by the weight changes.

This meta-analysis has shown a statistically significant association of SGLT2 inhibitors based therapy with adverse events. The number of patients experiencing genital or urinary tract infections was significantly higher in SGLT2 inhibitors treated groups than placebo treated groups. Both genital and urinary tract infections were more common among females than males [40]. However, the proportions of patients with severe adverse events, hypoglycemic events and discontinuation of medication because of adverse events in SGLT2 inhibitors treated groups were not different from placebo treated groups. Similarly, previous meta-analyses reported an increased risk of urinary and genital tract infections with dapagliflozin without a significant increase in hypoglycemic events [13,14]. Moreover, a study of women with type 2 diabetes has established the significant association of canagliflozin therapy with vulvovaginal candidiasis [41].

As limitations, firstly heterogeneity testing has revealed the presence of significant inconsistency among the included studies. But, the uncertainty of the results from this meta-analysis did not appear increased. This is because; sensitivity analysis by removing any of the study with a specific dose from the analysis confirmed the stability of the overall values. The possible explanation for the significant heterogeneity could be: the variation in the patients’ antidiabetic agents experience, the variation in the SGLT2 inhibitors regimens, and the difference in baseline demographic and clinical characteristics of the patients recruited.

Secondly, a pooled data from phase 2b and 3 clinical trials has shown an increased incidence of cancer and hepatotoxicity with dapagliflozin [42]. This meta-analysis did not have any evidence to rule out or to share these concerns. That is, further analyses were not conducted to determine the association of SGLT2 inhibitors with rare adverse events like cancer development and hepatotoxicity. Meta-analysis of rare adverse events, using studies that were not primarily designed to test adverse events, can yield misleading information [43]. Thirdly, since all the included studies were sponsored by pharmaceutical companies, the findings of studies could be biased by business interests. Lastly, the number of studies on canagliflozin, ipragliflozin and empagliflozin that were included in this meta-analysis is very small. Thus, the pooled values of this meta-analysis may not reveal the clinical effect.

## Conclusion

In conclusion, in patients with type 2 diabetes, all studied doses of SGLT2 inhibitors either as monotherapy or in combination with other antidiabetic agents improved glycemic control consistently. Furthermore SGLT2 inhibitors were associated with a significant reduction in body weight and blood pressure. However, a small percentage of patients suffer from genital and urinary tract infections. Yet, SGLT2 inhibitors appear to be safe as the number of hypoglycemic events and the number of patients who discontinued therapy were similar between SGLT2 inhibitor treated groups from placebo treated patients. Finally, further investigations with long term duration of therapy are needed to establish the safety and efficacy of SGLT2 inhibitors.

## Competing interests

The authors declare that they have no competing interests.

## Authors’ contributions

ABe: conceived and designed the study, conducted the analysis, wrote the manuscript and participated in the literature search, study selection and data abstraction. ABa: reviewed and edited the manuscript and participated in the literature search, study selection and data abstraction. Both authors read and approved the final manuscript.

## Pre-publication history

The pre-publication history for this paper can be accessed here:

http://www.biomedcentral.com/1472-6823/13/58/prepub
